# Factors influencing special education teachers’ self-efficacy to provide transitional services for students with disabilities

**DOI:** 10.3389/fpsyg.2023.1140566

**Published:** 2023-08-17

**Authors:** Sara Binammar, Aljazi Alqahtani, Ghaleb Hamad Alnahdi

**Affiliations:** ^1^Department of Special Education, College of Education, Princess Nourah bint Abdulrahman University, Riyadh, Saudi Arabia; ^2^Department of Special Education, College of Education, Prince Sattam bin Abdulaziz University, Alkharj, Saudi Arabia

**Keywords:** transition services, teachers’ self-efficacy, attitudes, students with disabilities, factors

## Abstract

This study examined factors that may influence the self-efficacy level of special education teachers in delivering transition services to students with disabilities. Five independent variables were examined: attitudes, preparation level, teaching experiences, academic degree level, and level of available resources. The current study sample comprised 231 intermediate and secondary special education teachers in the city of Riyadh, Saudi Arabia. The results showed that attitudes are the best predictor of teachers’ self-efficacy to provide transitional services for students with disabilities. Teacher preparation for transition services was the second most important variable that was positively associated with teachers’ self-efficacy.

## Introduction

An inclusive approach to education means that each individual’s needs are taken into account and that all learners participate and achieve together (UNESCO). Inclusive education has been considered a common policy worldwide that emphasizes equality in education for all children in mainstream settings (Alnahdi, 2020). In a similar vein, in Saudi Arabia, several policies have been developed to support the equal rights of individuals (i.e., students) with disabilities for obtaining free and appropriate education (Alquraini, 2011). For instance, the regulation of the Authority for the Care of People with Disabilities issued by the Council of Ministers stipulated policies as well as the provision of necessary care and rehabilitation for individuals with disabilities. This regulation also imposes the improvement of services provided to such individuals in terms of education, qualification, treatment, provision of work opportunities, facilitating access to public facilities, and enhancement of their position in society. In this context, an individual educational plan (IEP) was developed to reflect the different characteristics of individuals with disabilities and various methods appropriate for each group (Al Thabit, 2016). The IEP covers numerous services, including transition services.

The Individuals with Disabilities Education Act (Johnson, 2005) defines transition services as a “coordinated set of activities for a child with a disability…. that focused on improving the academic and functional achievement of the child with a disability to facilitate the child’s movement from school to postschool activities, including post-secondary education, vocational education, [and] integrated employment” (Johnson, 2005). In the outlined scenario, delivering transition services requires specialized team, including special education teachers. Although this is not always the case, special education teachers may differ in their provision of sufficient transition services. In other words, teachers’ success in delivering transition services may be subjected to their conviction regarding their abilities, which is known as self-efficacy.

Self-efficacy refers to individuals’ belief in their “capabilities to organize and execute the courses of action required to produce given attainments” (Bandura, 1977, p. 3). Individuals with high self-efficacy are distinguished based on their ability to accept high responsibility, high energy, logical thinking, master of difficult tasks, work toward achieving goals, and their ability to withstand pressure (Denzine et al., 2005; Tschannen-Moran and Hoy, 2007; Peebles and Mendaglio, 2014; Zee and Koomen, 2016). Self-efficacy is influenced by various factors, such as attitudes, which are defined as “individuals’ opinions, or/and how they feel, think, or act regarding something and/or someone” (Attitudes, 2019). This association is unsurprising due to “an assumption that behaviors are primally determined by individuals’ perceptions in the immediate situation” (Fazio, 1986, p. 207).

Association between teachers’ self-efficacy and teachers’ attitudes has been widely noted (Sharma et al., 2012; Alnahdi, 2020; Alnahdi and Schwab, 2021; Yada et al., 2022). Teachers’ self-efficacy is defined as their “judgment of his or her capabilities to bring about desired outcomes of students engagement and learning, even among students who may be difficult or unmotivated” (Tschannen-Moran and Hoy, 2001, p. 783). Numerous scholars have examined the correlation between teachers’ self-efficacy and their attitudes. On the one hand, many studies have found that self-efficacy positively influences teachers’ attitudes. Özokcu (2018) examined the correlation between the attitudes of 1,163 special education teachers and their self-efficacy and showed a positive correlation between teachers’ attitudes and their self-efficacy level. On the other hand, some studies have found that teachers’ attitudes can influence their self-efficacy. Alnahdi and Schwab et al. (2020) showed a positive correlation between teachers’ attitudes and their self-efficacy level. More positive attitudes in teachers indicate that they will be more positively reflected in their self-efficacy level. Therefore, to determine whether teachers’ self-efficacy influences their attitudes or their attitudes are influenced by their self-efficacy, the correlation should be examined.

Despite variations in the sequence of teachers’ self-efficacy and/or attitudes, both are key components that influence the competence and performance (i.e., provided transition services) of special education teachers. This may not be surprising due to the extent to which special education teachers have positive attitudes as well as being convinced regarding their abilities; this may help to provide more sufficient and effective transition services. Regardless of this importance, few international and national studies have examined self-efficacy in special education teachers in their endeavors to deliver transition services. Wolfe et al. (1998) identified teachers’ attitudes toward the transition of students with disabilities, and concluded that teachers had different attitudes toward transition services. Additionally, Alnahdi (2013) identified the attitudes of teachers in intellectual education programs toward transition services from school to work for students with mild mental disabilities. The study sample comprised 369 teachers involved in the intellectual education programs in the city of Riyadh. The results show that teachers had positive attitudes toward the importance of transition services for students with mental disabilities. Furthermore, Alnahdi (2014) explored the teachers’ perceptions regarding their willingness to provide transition services, and revealed negative perceptions among teachers regarding their abilities to deliver transition services as well as qualifications they received in preparation programs.

### Factors influencing special education teachers’ self-efficacy

Studies worldwide have shown notable interest in examining the correlation between teachers’ self-efficacy and certain factors (personal experiences, academic degree, etc.). Leyser et al. (2011) identified the effect of numerous years of experience on teachers’ self-efficacy and showed the effect of years of experience on improvements in self-efficacy. Additionally, Peebles and Mendaglio (2014) examined the correlation between educational experience and self-efficacy, finding that teachers with more experience with people with disabilities have notably higher self-efficacy than those who do not have the relevant experience.

Furthermore, other studies examined the correlation between teachers’ self-efficacy and academic degree level. Nuri et al. (2017) focused on 70 special education teachers to identify self-efficacy with relation to numerous variables—gender, academic degree, daily work hours, and daily numbers of teachers. Their findings show the differences attributable to the academic qualification variable in favor of postgraduate studies. Moreover, Al-Fakhoury (2018) attempted to identify the correlation between psychological stress levels and teachers’ self-efficacy for blind students in light of the variables of academic qualification, years of experience, income level, and vision status. The results showed differences in the averages of the proficiency scores caused by the academic degree variable for the benefit of the bachelor’s and master’s degree.

Additionally, other studies examined the effect of training courses on teachers’ self-efficacy. Kormos and Nijakowska (2017) examined the self-efficacy level and teachers ‘attitudes toward the use of comprehensive educational practices before and after the training course. The sample comprised 752 teachers, and the results indicate improvement in teachers’ self-efficacy after the training course. Similarly, Chao et al. (2017) examined the effect of a training course on teachers’ self-efficacy in terms of improving teaching, learning, and classroom management strategies in regular schools in Hong Kong. The study sample comprised 347 teachers, and the results confirmed that the training course led to an increase in teachers’ self-efficacy levels.

Moreover, numerous studies have addressed the impact of resource availability level on self-efficacy Avramidis and Norwich (2002) conducted a literature review of teachers’ attitudes toward inclusion and resource availability in the school. Their results showed a positive correlation between teachers’ perceptions regarding resource availability and teachers’ self-efficacy level to perform the tasks expected of them. Despite the usefulness of this construct as an indicator of teachers’ self-efficacy and attitudes, scant research has assessed the correlation of special education teachers’ self-efficacy and influential factors in transition services.

## The current study

The current study examined the factors affecting the self-efficacy levels of special education teachers in delivering transition services to students with disabilities in Riyadh. To this end, we investigated the predictors of teachers’ self-efficacy. Based on the literature, we posited that teachers’ attitudes, training courses, academic degree, resource availability level, and teaching experiences would predict special education teachers’ self-efficacy and thus formulated the following hypotheses:

*H1*: There is a positive association between teachers’ attitudes toward transition services and their self-efficacy level.

*H2*: There are statistically significant differences in the self-efficacy level of special education teachers that can be attributed to the years of experience, academic degree variables, training courses, and resource availability level.

Therefore, this study aims to answer the following questions:

Is there a positive association between teachers’ attitudes toward transition services and their self-efficacy level?

Are there statistically significant differences in the self-efficacy level of special education teachers that can be attributed to the years of experience, academic degree variables, training courses, and resource availability level?

## Methods

### Design of the investigation.

This study was designed to test some of the hypotheses found in some previous studies about the relationship of teachers’ self-efficacy with other variables. Self-efficacy in other studies dealt with it in relation to work in inclusive education environments in general in some cases. In this study, the focus was on the teacher’s efficiency in applying transitional plans and their relationship to some other variables.

### Participants

The study sample comprised special education teachers in the intermediate and secondary stages in Riyadh (*n* = 878). The study sample was 238 based on the recommendation of the G*Power software, after considering a set of study variables and the number required to verify the existence of a statistical effect on the variables. Ethical approval was obtained from the IRB Committee at Prince Sattam Bin Abdulaziz University. Following this, the survey scales were applied to an exploratory sample total of 50 participants, including 28 female teachers and 22 male teachers. This pilot study aimed to examine the psychometric characteristics of instruments and check their validity before applying them to the entire sample. The electronic version of the survey was also distributed through a WhatsApp link, as well as Twitter. A total of 238 special education teachers provided survey responses. This is done through a teacher cooperating with the research team who sends an electronic copy of the scale to his/her fellow teachers in the school. In sum, the snowball technique was used to reach the full sample of this study, with help of some teachers working in schools.

### Measures

All measures adopted in this study were in Arabic except for the scale of the resources (Alnahdi et al., 2021) which was translated to Arabic from English. Back-translating approach was followed to reach the final Arabic version (Beaton et al., 2000). To develop the instrument used in this study, items were adapted from various scales: the parent participation scale (Almalki et al., 2021); self-efficacy scales (Abu and Hatem, 2019; Al-Hawaiti, 2019); self-efficacy scale for teachers in inclusive education (TEIP; Sharma et al., 2012; Alnahdi, 2019, 2020), teachers’ attitudes toward transition services scale (Alnahdi, 2013); and resources in the school scale (PRQ; Alnahdi et al., 2021). The questionnaire included two main sections. The first section asked demographic questions (gender, age, educational qualification, marital status, name of their school, the stage they teach, and specialization). The second section includes the following: the self-efficacy scale, which was developed to measure teachers’ self-efficacy level, contains four items that measure teachers’ beliefs regarding their ability to plan for transition services, two items to measure their beliefs regarding their ability to implement transition services, and two items to measure their beliefs regarding their ability to work collectively in delivering transition services. The attitudes scale included four items that measure teachers’ attitudes toward transition services from Alnahdi’s (2013) questionnaire. The resources scale included 10 items to measure resource availability in the school from the perspective of teachers (see the PRQ scale by Alnahdi et al., 2021). The preparation subscale includes two items to measure whether teachers were prepared for transition services (Alnahdi, 2013).

### Data analysis

Quantitative data were statistically analyzed using SPSS software. Different statistical analyses were used to address research questions. First, the reliability coefficients of the scales were examined. Cronbach’s α values were calculated to examine the internal consistency for each subscale. In addition, descriptive statistics, such as mean and standard deviation, were calculated to understand the data. Second, path analysis was used to examine the best model (with different predictors) to explain the observed data using AMOS software. After screening the data, and the normality was supported by checking kurtosis values for all variables, looking for values of 3 or less (Stevens, 2009). Two models were tested to determine which model adequately explained variations in the observed data.

## Results

Table 1 reports the examination of the internal consistency of all scales used in this study in two steps. First, this was conducted with the pilot sample (*N* = 50) to ensure that adequate psychometric properties appeared in this study. Second, reliability statistics were checked for the whole sample with values of Cronbach’s α, McDonald’s ω, and Guttman’s λ6. In both steps, the values of α were in the very good range, from 0.75 to 0.98, as an indicator of good reliability (George and Mallery, 2003). In addition, composite reliability was calculated (see Table 1), and the convergent validity was supported by having values above 0.5 on the average variance extracted (AVE) on all scales (Fornell and Larcker, 1981; Hair et al., 2010). In addition, confirmatory factor analysis was conducted for the three scales (SE, ATT, and PRQ) and the results showed good indicators as regards the fit indices for these scales (see Appendix 1). For example, a Comparative Fit Index (CFI) higher than 0.95, a Goodness of fit index (GFI) higher than 0.95, and Tucker-Lewis Index (TLI).

**Table 1:** Reliability statistics.

	Pilot sample Cronbach’s α (N = 50)	Full sample Cronbach’s α	McDonald’s ω	Guttman’s λ6	CR	AVE	Items
SE	0.964	0.983	0.982	0.988	0.980	0.0834	10
ATT	0.899	0.904	0.751	0.824	0.897	0.687	4
PRQ	0.947	0.950	0.950	0.971	0.947	0.644	10

SE = self-efficacy, ATT = attitudes scale, PRQ = resources scale, CR = composite reliability, AVE = average variance extracted.

Table 2 shows participants’ means on all four scales in this study. In general, participants expressed a good level of self-efficacy to conduct transition services for students with disabilities (*M* = 3.83, *SD* = 0.92). Attitudes toward transition services level were good and above the theoretical mean (*M* = 4.13, *SD* = 0.79).

**Table 2:** Means and standard deviations.

Demographical Variables		SE	ATT	PRQ	PRE
Gender	Female (*N* = 114)	3.72 (0.86)	4.09 (0.79)	3.12 (0.92)	2.84 (1.0)
	Male (*N* = 117)	3.93 (0.96)	4.17 (0.80)	2.94 (1.1)	2.58 (1.0)
School level	Middle (*N* = 121)	3.63 (0.95)	4.03 (0.85)	3.07 (1.0)	2.89 (1.1)
	High (*N* = 110)	4.05 (0.84)	4.24 (0.71)	3.00 (1.0)	2.51 (0.98)
Total (*N* = 231)		3.83 (0.92)	4.13 (0.79)	3.03 (1.0)	2.71 (1.0)

SE = self-efficacy scale, ATT = attitudes scale, PRQ = resources scale, PRE = preparation in transition.

Next, structural equation modelling (SEM) was used to compare the fit indices of three different models against the observed data in this study. In these analyses, different indicators for fit are used to select the best model in explaining the observed data; thus, a value of RMSEA less than 0.1 (Browne and Cudeck, 1992) would indicate a reasonable error of approximation. In addition, the NFI and Tucker-Lewis coefficient (TLI) > 0.9 indicate an acceptable fit (Bentler and Bonett, 1980), and a value of χ^2^/*df* around 3 would be a good indicator (Marsh and Hocevar, 1985; Kline, 1994). Moreover, examining the fit indices for the models, a non-significant correlation of variables with self-efficacy would be a reason for removing a variable from the next model.

**Figure 1:**
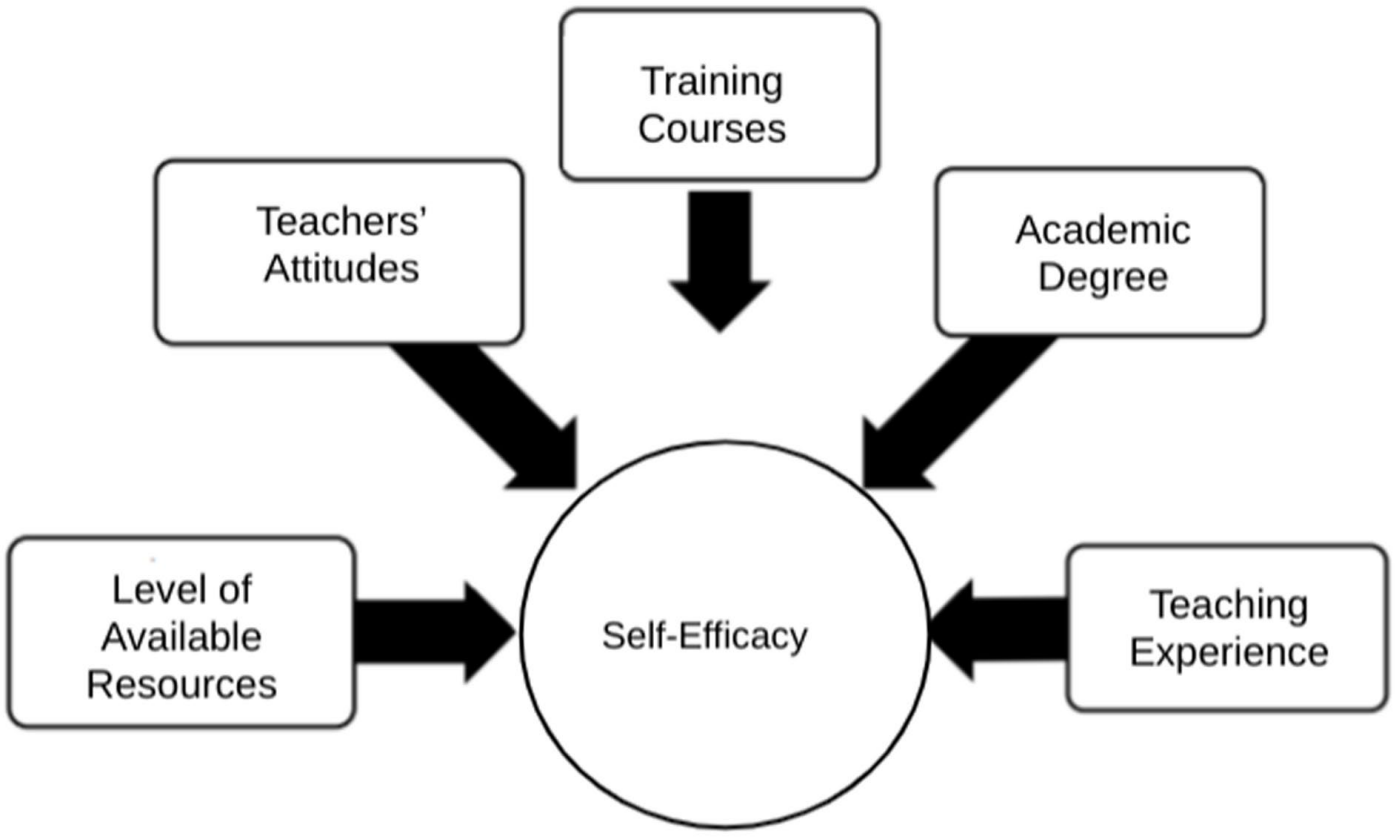
Variables that may affect teachers’ self-efficacy (study hypotheses).

In the first model (M1), eight variables were used to predict teachers’ self-efficacy levels. These variables were attitudes, preparation (at the university level), gender, degree, training, years of teaching experience, and two variables related to the school: resource availability at the school and school level (Figure 1). The SEM analysis results showed that three variables (degree, gender, and training) were not significant at *p* < 0.05 and were thus removed from the next model. In the next model (M2), four variables were retained. The lowest latent variable in was PRQ (standardized coefficient), with 0.110.

**Figure 2:**
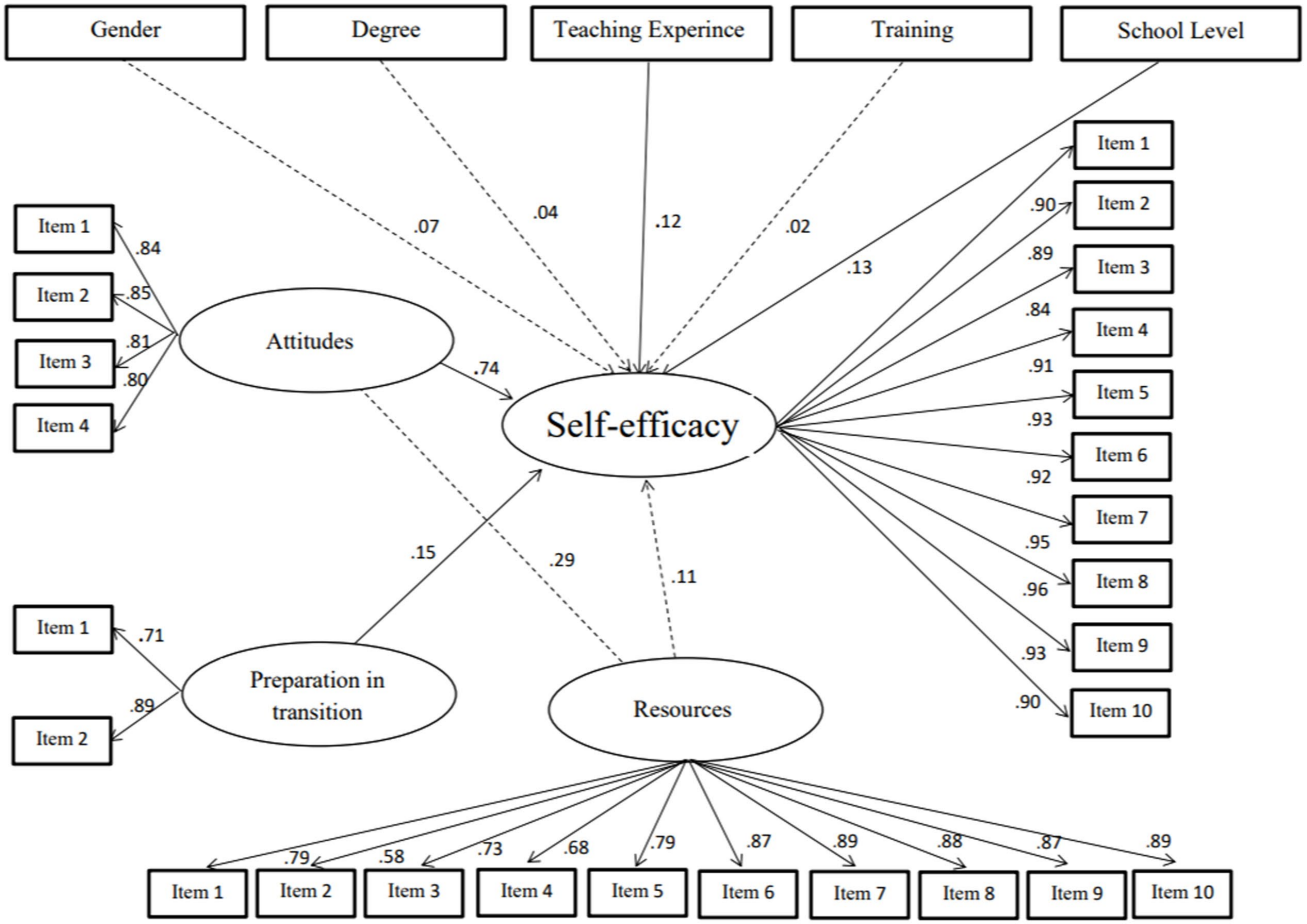
Model 1.

**Figure 3:**
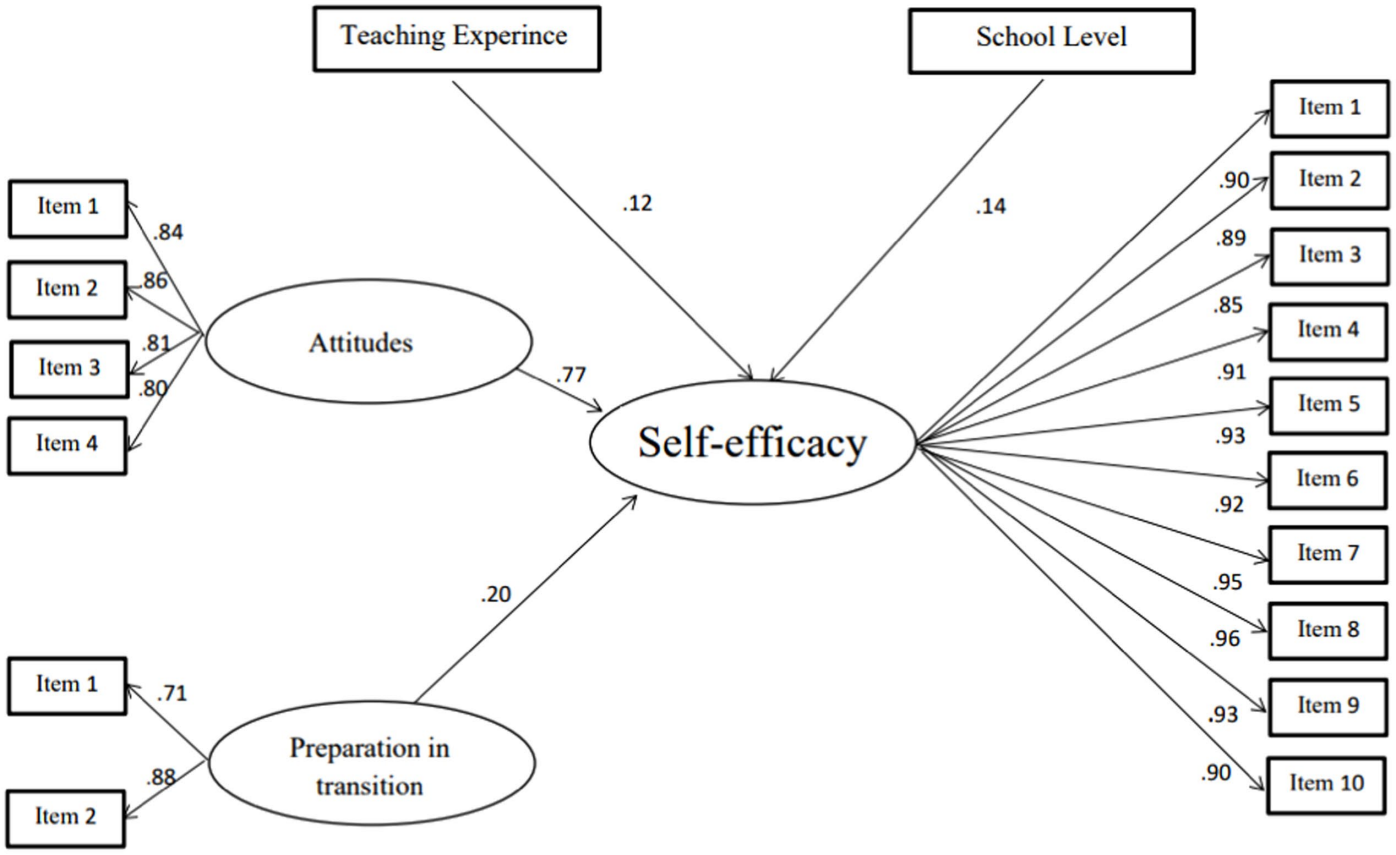
Model 3.

The last model (M3) showed the best model with best-fit indices, such as CFI = 0.96, and χ^2^/*df* < 3. In addition, the Akaike information criterion (AIC) decreases from 1545.44 to 436.75 and the Bayes information criterion (BIC) decreases from 1879.35 to 622.64. All latent variable associations in this model are significant at *p* < 0.01. This model includes attitudes, preparation, school level, and teaching experience (Figures 2, 3). Attitude is the variable that has a highly positive association with participants’ self-efficacy level 0.77.

In summary, attitude is the most important variable in relation to teachers’ self-efficacy, and other variables, such as preparation at the university level and years of experience, are associated with the level of self-efficacy (Table 3).

**Table 3:** Fit indices for all three models.

	CMIN (*df*)	CMIN/*df*	RMSEA	CFI	TLI	NFI
M1	1351.44** (399)	3.4	0.1	0.88	0.86	0.84
M2	1095.32** (315)	3.5	0.1	0.89	0.88	0.87
M3	328.75** (117)	2.8	0.89	0.96	0.95	0.936

M1 = model 1 with eight variables, M2 = second model with five variables, M3 = last model with four variable, RMSEA = Root Mean Square Error of Approximation, CFI = Comparative Fit Index (Bentler and Bonett, 1980), TLI = Tucker-Lewis coefficient, NFI = Normed Fit Index.

** = *p* value is 0.01.

## Discussion

Special education teachers’ self-efficacy is seemingly associated with providing transition services. In the current study, special education teachers indicated a good level of self-efficacy in providing transition services for students with disabilities. This finding aligns with that of Schwab and Alnahdi (2020) that a moderate-to-high correlation was noted between teachers’ self-efficacy and use of inclusive teaching practices. In a similar vein, this findings aligned with that of Alnahdi and Schwab (2021) that Saudi Arabian special education teachers expressed high self-efficacy levels.

In the current study, attitudes were a key variable that is positively associated with teachers’ self-efficacy. This finding aligned with that of Sharma et al. (2012) confirming a positive relationship between positive attitudes toward inclusive education and high self-efficacy levels. In a similar vein, Alnahdi and Schwab’s (2021) findings revealed that teachers’ attitudes toward inclusive education was the strongest predictor of teachers’ self-efficacy. Based on this scenario, the connection between attitudes and self-efficacy seemed to predict delivered transition services to some extent. Schwab and Alnahdi (2020) assured that teachers’ attitudes toward inclusive education may predict their self-efficacy beliefs in inclusive education, and the latter was shown to predict teachers’ use of inclusive teaching practices.

Furthermore, the study findings showed the positive impact of teacher preparation on their self-efficacy, as well as how teaching experiences can be slightly associated with their self-efficacy. This finding aligned with that of Peebles and Mendaglio (2014) thereby emphasizing the importance of coursework, as well as field experience, in developing self-efficacy. The study participants gained high self-efficacy after the inclusion course. In a similar vein, Burton and Pace’s (2009) findings illustrate that both coursework and direct experience significantly increased teachers’ self-efficacy for teaching diverse learners.

Based on the study findings, two key arguments can be made. First, teachers’ self-efficacy may be developed through training and coursework. Second, teachers’ self-efficacy may help in providing good education (i.e., transition services), thus students’ achievements may be improved as well. Lee et al.’s (2011) findings highlight the importance of well-designed and effective teacher education programs that provide high-quality education. Likewise, Tschannen-Moran and Hoy (2007) noted positive relations between teachers’ self-efficacy, their success in the classroom, and student achievement levels. Furthermore, Zee and Koomen’s (2016) findings emphasized the correlation between teachers’ self-efficacy and students’ academic adjustment.

The aforementioned argument highlights the importance of teachers’ preparation programs. Nonetheless, this may not be the case in the Saudi context. Currently, there is conflicting evidence regarding the skill levels of Saudi special education teachers. Many teachers have denoted their dissatisfaction with the training program in Saudi Arabia. A study conducted by Alnahdi (2014) indicated that teachers had negative impressions of their training programs. Likewise, Al Thabit (2016) noted that special education teachers indicated that they were not well-prepared. In a similar vein, Hussain’s (2009) findings showed that Saudi special education teachers denoted many aspects of their teaching to be insufficiently addressed in the coursework provided by the Department of Special Education in Saudi universities. Therefore, studies focusing on the Saudi context have attempted to address the gap in teachers’ preparation programs. Al-Atwi and bint Muhammad Ababa (2020) recommended the importance of preparing and providing special education teachers with training courses to enhance their performance in relation to delivered transition services.

## Conclusion and implications

This study revealed key findings regarding teachers’ self-efficacy in the field of special education, particularly in transition services. The current study highlights correlations between several factors (i.e., attitudes, preparation programs, teaching experiences, academic degree level, and resource availability) and self-efficacy from the perspectives of Saudi special education teachers. Interestingly, teachers’ attitudes were noted as the main variable that is positively associated with their self-efficacy to conduct transition services. Additionally, teachers’ preparation programs as well as teaching experiences were also important variables associated with teachers’ self-efficacy.

As indicated earlier, few national and international studies have investigated self-efficacy from the perspective of special education teachers. The current study helped to address this gap in the context of Saudi special education teachers expressing their perspectives regarding transition services. Furthermore, to the best of our knowledge, research has yet to examine the relationship between self-efficacy from the perspectives of special education teachers in primary-intermediate mainstream schools in the Saudi context. This study contributes to the body of international and national literature in the field of special education, particularly transition services in a rarely addressed context (Riyadh, Saudi Arabia). The study results provide key insights for stakeholders, schools, and teachers. Therefore, there are several implications to consider.

Another finding highlights the correlation between attitudes and self-efficacy. As presented earlier, teachers’ attitudes take the lead in the cyclical process that eventually influences their self-efficacy. Hence, stakeholders must consider attitudes when recruiting teachers for transition services. This consideration is truly important because teachers’ attitudes significantly impact their self-efficacy, performances, and potential for success in inclusive settings (i.e., delivered translon services). Moreover, stakeholders should determine various methods that may increase teachers’ positive attitudes. The implementation, adoption, and development of new polices and/or standards may help to develop special education teacher’s attitudes. For instance, implementing CEC standards is one significant method that will implicitly aid in the development of special education teachers’ attitudes. CEC standards are defined as “what beginning special education teachers need to know and be able to practice safely and effectively” (Council for Exceptional Children, 2004, p. 2). CEC standards can be considered a key guide providing special education teachers with necessary aspects for dealing with diversity of students with disabilities. This is unsurprising because “it is appropriate to ask how these teachers will develop effective teaching and assessment strategies while dealing with the diversity of issues involving parents, colleagues, and students. It seems unlikely that any one of these challenges could be met with no system of evaluation in place or any guidance from professional standards available, by which to judge their performance” (Binammar, 2020, p. 660). Consequently, the adoption of these standards may lead to improved outcomes for special education teachers in terms of positive attitudes, high self-efficacy, and delivered transition services. Consequently, this may lead to improved learning outcomes among students with special needs.

Likewise, the current study illustrates correlations between teacher preparation programs and self-efficacy. This finding emphasizes that the role of teacher preparation programs becomes more critical specifically in the Saudi context. Overarching implications for stakeholders are important for developing special education teachers’ programs, specifically when designing transition services programs. Stakeholders should carefully consider the combination of both course work and direct experiences. Emphasis should also be placed in providing special education teachers with the required knowledge, skills, instructions, and strategies for managing behaviour. Importantly, teachers’ preparation programs should focus on helping special education teachers applying and transferring theoretical knowledge to a practical one in inclusive settings. Thus, self-efficacy may increase and eventually, sufficient transition services may be delivered. Lee et al. (2011) clarified that teacher preparation programs must ensure the provisions of meaningful, realistic, and challenging experiences for teachers to be capable of delivering effective and efficient instruction to diverse students. This implication is important based on the current shortcomings mentioned earlier regarding teacher’s preparation programs. Furthermore, providing training courses should help special education teachers with developing positive attitudes. Avramidis et al. (2000a,b, p. 291) stated that “providing extensive opportunities for training for prospective teachers in inclusive settings may also support the development of confidence and competence…teachers may not hold negative attitudes. Therefore, the boost of teacher self-efficacy is mainly a matter of teacher training.”

Additionally, another important finding in the current study was the association between teaching experiences and self-efficacy. This finding implied the key role that schools must undertake. Schools should consider the importance of providing special education teachers with the necessary information, sources, and skills. Providing this information and sources will increase the positive experiences of special education teachers and eventually enhance their efficacy level, thus ensuring that sufficient transition services can be delivered.

## Recommendations for future research

Research has yet to thoroughly examine teachers’ attitudes and the correlation to transition services in the Saudi context. Future research should examine factors that can explain the variation in special education teachers’ attitudes, as well as their self-efficacy in relation to delivered transition services. Gender differences are also a key factor that must be considered in future studies, particularly in countries where schools are separated by gender, as they are in Saudi Arabia. Additionally, it is recommended that a comparative study be conducted of special education teachers’ self-efficacy in relation to delivered transition services in two different settings: mainstream and private schools. The current study focused on primary and secondary mainstream schools. It would be interesting to see if delivered transition services practices differ in other settings, which may expose different perspectives regarding teachers’ attitudes and their self-efficacy in relation to transition services. Any differences noted between the two groups can yield key insights regarding methods for enhancing special education teachers’ self-efficacy. In addition, it is important to study the extent to which self-efficacy reflects significantly on teachers’ practices to deliver transition services, by collecting information about self-efficacy as well as information about practices.

## Limitations

There are some limitations that should be considered in this study. The first limitation regards methodology in the current study. Only an online survey was distributed among special education teachers, which may include a sample of teachers that may not be representative of all teachers. However, a sample with more than 200 teachers could be considered an adequate sample for the statistical analysis conducted in this study. The second limitation is related to the sampling technique (i.e., convenience sampling) used in this study. Third, participants in the current study were from one city in Saudi Arabia (Riyadh). Therefore, the study findings may not be representative of the total population of special education teachers in Saudi Arabia. Conducting similar studies and combining their results may help to generalize the data to the total population. In addition, another limitation is the social desirability effect that could influence participants’ responses to the Questionnaire.

## Data availability statement

The original contributions presented in the study are included in the article/supplementary materials, further inquiries can be directed to the corresponding author.

## Ethics statement

The studies involving human participants were reviewed and approved by the IRB committee at Prince Sattam bin Abdulaziz University. Written informed consent for participation was not required for this study in accordance with the national legislation and the institutional requirements. Written informed consent was not obtained from the individual(s) for the publication of any potentially identifiable images or data included in this article.

## Author contributions

All authors listed have made a substantial, direct, and intellectual contribution to the work and approved it for publication.

## Funding

This study is supported via funding from Prince Sattam bin Abdulaziz University project number (PSAU/2023/R/1444).

## Conflict of interest

The authors declare that the research was conducted in the absence of any commercial or financial relationships that could be construed as a potential conflict of interest.

## Publisher’s note

All claims expressed in this article are solely those of the authors and do not necessarily represent those of their affiliated organizations, or those of the publisher, the editors and the reviewers. Any product that may be evaluated in this article, or claim that may be made by its manufacturer, is not guaranteed or endorsed by the publisher.

## Appendix 1

Fit indices from confirmatory factor analysis for the scales.

**Table:**

Index	PRQ-T	SE	ATT
Comparative fit index (CFI)	0.996	0.999	0.999
Tucker-lewis index (TLI)	0.995	0.999	0.998
Goodness of fit index (GFI)	0.995	0.999	0.999
Bentler-Bonett normed fit index (NFI)	0.995	0.999	0.998
Standardized root mean square residual (SRMR)	0.052	0.034	0.036
Bollen’s relative fit index (RFI)	0.994	0.999	0.996
Bollen’s incremental fit indesx (IFI)	0.996	0.999	0.999
Relative Noncentrality index (RNI)	0.996	0.999	0.999
